# MORAL ENHANCEMENT VIA DIRECT EMOTION MODULATION: A REPLY TO JOHN HARRIS

**DOI:** 10.1111/j.1467-8519.2011.01919.x

**Published:** 2011-11-17

**Authors:** Thomas Douglas

**Keywords:** moral enhancement, biomedical enhancement, moral education, emotion, freedom, John Harris

## Abstract

Some argue that humans should enhance their moral capacities by adopting institutions that facilitate morally good motives and behaviour. I have defended a parallel claim: that we could permissibly use biomedical technologies to enhance our moral capacities, for example by attenuating certain counter-moral emotions. John Harris has recently responded to my argument by raising three concerns about the direct modulation of emotions as a means to moral enhancement. He argues (1) that such means will be relatively ineffective in bringing about moral improvements, (2) that direct modulation of emotions would invariably come at an unacceptable cost to our freedom, and (3) that we might end up modulating emotions in ways that actually lead to moral decline. In this article I outline some counter-intuitive potential implications of Harris' claims. I then respond individually to his three concerns, arguing that they license only the very weak conclusion that moral enhancement via direct emotion modulation is sometimes impermissible. However I acknowledge that his third concern might, with further argument, be developed into a more troubling objection to such enhancements.

Recent history is littered with atrocities. The Nazi's Final Solution, Mao's Cultural Revolution, and recent genocide in Rwanda and the Balkans are but a few examples. Some of these atrocities can be attributed to a few evil individuals. But it is increasingly appreciated that many of humanity's greatest wrongs were made possible (and in some cases even perpetrated) by ordinary people who were just not as moral as they might have been.^1^ Holocaust historian Christopher Browning entitled his most well-known book about the Final Solution *Ordinary Men* in order to emphasize this fact.

Some ethicists, motivated in part by a concern to prevent humanity from replicating the atrocities of the 20th century, have suggested that their discipline needs to develop a new, more empirically informed approach, and to foster a moral climate that directs more attention to harms inflicted at a distance.^2^ Others have called for institutional reforms designed to promote morally better ways of thinking and behaving.^3^ The distribution of responsibilities through a bureaucratic and hierarchical power structure in Nazi Germany made it easy for ordinary people to allow, and in some cases contribute to, the annihilation of European Jewry. Perhaps there are different institutional structures that could have the opposite effect, making it easier, rather than harder, to think and do the right thing. We could think of such structures as external, institutional means for enhancing human morality.

In recent writings, some philosophers have entertained a parallel possibility: that we might also enhance moral capacities through internal, biomedical means (call this ‘biomedical moral enhancement’). Ingmar Persson and Julian Savulescu have argued that there is an urgent need for widespread biomedical moral enhancement.^4^ I have argued for a weaker conclusion: that, under certain conditions that may come close enough to obtaining, individuals may permissibly use biomedical technologies to morally enhance themselves.^5^ (I put forward this argument not because I think it is of great practical importance – perhaps rather few people would actually *want* to morally enhance themselves – but in order to highlight the implausibility of the popular view that engaging in biomedical enhancement *of any sort* is always morally impermissible.)

In arguing for this claim, I understood moral enhancement to consist in the acquisition of morally better motives. There are, of course, widely divergent views on what motives are morally good, and thus on what kinds of psychological changes would constitute moral enhancements of this sort. Some would hold that moral reasoning is the only morally good motive, while others might emphasize pro-social emotions such as sympathy. In an attempt to find some common ground, I suggested that moral enhancement might consist in the attenuation of counter-moral emotions: emotions that interfere with moral reasoning, sympathy, and all other plausible candidates for ‘morally good motives’. Exactly which emotions these are will vary across individuals and environmental circumstances, but I speculated that in some persons in some contexts, racial aversion and impulses to violent aggression might qualify. Thus, I suggested that biomedical moral enhancement might sometimes consist in the biomedical attenuation of these emotions. I had in mind the following sort of case:

[*The Biased Judge*] James is a district court judge in a multi-ethnic area. He was brought up in a racist environment and is aware that emotional responses introduced during his childhood still have a biasing influence on his moral and legal thinking. For example, they make him more inclined to counsel jurors in a way that suggests a guilty verdict, or to recommend harsher sentencing, when the defendant is African-American. A drug is available that would help to mitigate this bias.

On the basis of this schematic description, it seems possible that James' taking the drug would qualify as a biomedical moral enhancement, by my account.

## MORAL CONCERNS ABOUT MORAL ENHANCEMENT

The appeal of moral enhancement – whether through institutional or biomedical means – is not difficult to see. Moral enhancement might help to prevent atrocities, the threat of which grows as the human capacity to harm *en masse* increases.^6^ But it might also help to avert other less dramatic forms of harm, as the *Biased Judge* case suggests. Arguably, morally enhanced individuals would exhibit less prejudice, pollute less, do more to fight developing world disease and poverty, and be better friends and partners. Moral enhancement could therefore be instrumentally good: good as a means to some other good such as the prevention of harmful or wrongful conduct, or simply to the good of being moral. But it might also be thought to be intrinsically good. Perhaps acts of moral improvement are good in themselves.

Nevertheless, there is significant reluctance to countenance biomedical moral enhancement. Indeed, there may be even greater resistance to it than to other varieties of biomedical enhancement, such as the use of pharmaceuticals to augment the memory, attention, or sporting prowess of normal persons. One of the few published public opinion studies on biomedical enhancement found that, of nineteen traits that might be biomedically enhanced, respondents were *least* willing to enhance the most morally significant traits on the list: empathy and kindness.^7^ Within academic debate on the ethics of biomedical enhancement, this resistance is also evident. Though moral enhancement is seldom explicitly discussed in this literature, some opponents of biomedical enhancement have sought to illustrate their concerns by reference to interventions that could be construed as biomedical *moral* enhancements, such as methylphenidate (Ritalin) use in ill-behaved children.^8^

Resistance to biomedical moral enhancement among bioconservative writers and the general public is unsurprising. In both groups, concern about biomedical enhancement has traditionally focussed on its potential to alter fundamental traits – traits central to our identity or personality or humanity – and moral characteristics are fundamental traits *par excellence*.^9^ However, opposition to biomedical moral enhancement has now come from an unexpected quarter. In a recent article in this journal, John Harris – until now one of the most consistent and enthusiastic proponents of biomedical enhancement – advances a rich, sustained, and multi-faceted critique of recent work sympathetic to biomedical *moral* enhancement. He seeks to undermine Persson and Savulescu's case for biomedical moral enhancement, and he also presents an independent case against it. In this article, I wish to respond to this latter case, which is directed largely at my own earlier work.

In fact, it is somewhat misleading to say that Harris offers a case against biomedical moral enhancement, for he is not opposed to all biomedical moral enhancements, or even to all that fit my schematic characterization: moral enhancement through the attenuation of counter-moral emotions. For example, he would not object to a biomedical intervention that mitigated xenophobia *by* enhancing general cognition, thereby reducing the tendency to hold false stereotypic beliefs. He puts such interventions in the same (unproblematic) category as ‘bringing up children to know the difference between right and wrong’ or ‘general education, including self education, wide reading and engagement with the world’.^10^ What he objects to is the enhancement of morality via the *direct* biomedical modulation of emotions – that is, without, as an intermediate step, increasing the accuracy of cognitive processes (such as reasoning) or cognitive states (like beliefs).^11^ He would be concerned, for example, about the use of a drug that directly attenuated xenophobia, rather than by correcting the false beliefs on which it may be based.

Harris' concerns are also not limited to biomedical moral enhancement; they apply, quite generally, to moral enhancement via the direct modulation of emotions. One way in which emotions might be directly modulated is through the use of pharmaceuticals and other biomedical technologies, but, as we shall see, there are also non-biomedical means of directly modulating emotions, and these fall within the scope of Harris' critique.

I believe that biomedical moral enhancement could be morally permissible even when it consists in the direct, biomedical modulation of emotions. I also believe that traditional, non-biomedical varieties of moral enhancement could be permissible when they operate directly on emotions. So in this paper, I wish to respond to Harris' case against direct emotion modulation as a means to moral enhancement. That case consists of three concerns. First, that direct means would be ineffective in modulating the relevant emotions. Second, that direct modulation of emotions would invariably come at an unacceptable cost to our freedom. And third, that we might end up modulating emotions in ways that actually lead to moral *decline*.

In responding to these concerns, I will understand moral enhancements to be interventions that will expectably leave an individual with more moral (*viz*., morally better) motives or behaviour than she would otherwise have had.^12^ I will use ‘noncognitive moral enhancement’ to refer to moral enhancement achieved through (a) modulating emotions, and (b) doing so directly, that is, not by improving (*viz*., increasing the accuracy of) cognition.

## A GENERAL RESPONSE

Before tackling Harris' concerns individually, I wish to briefly offer a general response to them.

As noted above, we already have non-biomedical means of pursuing noncognitive moral enhancement. One example would be stimulus avoidance. Consider the case of a serial philanderer who, under the influence of unwanted sexual desires, repeatedly violates his relationship commitments, thus harming those near to him. Arguably, one way for such an individual to morally enhance himself would be to avoid situations that tend to elicit the unwanted sexual desires. Another way might be to increase the personal costs to himself of seeking out such situations in the future. He might, for example, tell his friends that he will no longer go to such-and-such bar, thus guaranteeing embarrassment and loss of face if he is seen there.

These instances of stimulus avoidance plausibly operate through the direct modulation of emotional states. They need not correct or prevent any cognitive errors; nevertheless, by removing certain cues from the environment, they may prevent unwanted emotions from being elicited. Of course, they may be *motivated* by reasoning, or other cognitive processes, but that is also true of paradigmatic examples of noncognitive moral enhancement: for example, the direct pharmaceutical modulation of emotion. The distinctive feature of noncognitive moral enhancement is that it is not achieved by cognition-improving *means*. This is plausibly the case with stimulus avoidance. However, stimulus avoidance can, intuitively, be morally permissible, and indeed morally desirable. Perhaps it would be *better* if the philanderer simply resisted his temptations to cheat, or used other, cognitive means of changing his behaviour. But assuming that all superior methods have been exhausted, stimulus avoidance would be a *prima facie* acceptable strategy.

Here is a further possible example of direct emotion modulation via non-biomedical means. Suppose I believe that I ought to be more moved by the plight of the global poor, and ought to do more to help them. However, I have trouble drumming up much sympathy for them. To remedy this, I set up my television so that it regularly displays disturbing and graphic images of the effects of poverty, though for such brief periods that I do not consciously recognize them. Nevertheless, through subliminal effects, the images increase my feelings of sympathy. This is plausibly (though not uncontroversially) a moral enhancement, and it also plausibly operates via the direct modulation of emotions, not via cognitive improvement. It seems to fall within the scope of Harris' critique. Yet again, though some might find it somewhat troubling, it is doubtful that my action here is morally impermissible.

To the extent that Harris' concerns are meant to support a *general* indictment of noncognitive moral enhancement, they seem to be at odds with our intuitions about at least some cases. Some kinds of noncognitive moral enhancement *seem* permissible. This poses a significant challenge for Harris. On the one hand, he can accept, in accordance with common sense, that *some* variants of noncognitive moral enhancement are permissible. But then, unless he can say something substantive about when or how often it is impermissible, he will be left with a rather weak conclusion: that noncognitive moral enhancement is *sometimes* morally impermissible. On the other hand, he can maintain that noncognitive moral enhancement is always impermissible. But in that case he surely owes us an explanation of why we should ignore our intuitions to the contrary.

Against the backdrop of these general worries about Harris' project, let us now turn to consider his specific concerns about noncognitive moral enhancement. In assessing his concerns, I will be particularly interested to determine whether they can sustain any conclusion stronger than that noncognitive moral enhancement is *sometimes* morally impermissible.

## THE FIRST CONCERN: INEFFECTIVENESS

I have previously suggested that moral enhancement might be achieved by attenuating certain counter-moral emotions. Somewhat more tentatively, I also suggested that, in some people in some circumstances, racial aversion and impulses towards violent aggression might count as counter-moral emotions. So I speculated that attenuating racial aversion or violent aggression might sometimes qualify as a moral enhancement.

Harris does not dispute this. But he does dispute another idea (one that I have not previously endorsed but now wish to defend): that the attenuation of emotions such as racial aversion could be achieved through direct means:

it seems unlikely that … an aversion to certain racial groups, or to one or more gender or sexual orientation is simply a ‘brute’ reaction, a sort of visceral response, as perhaps is an aversion to spiders. Rather it is likely to be based on false beliefs about those racial or sexual groups and or an inability to see why it might be a problem to generalise recklessly from particular cases. In short prejudice, as well as rationality, usually has cognitive content and often makes factual claims. Beliefs with cognitive content are for example beliefs that X is true or Y is false, that A is a danger and B is not, that C is good and D is evil, they are explained by the people that have them in terms of beliefs and ideas, including beliefs about facts which may be, and therefore can often be shown to be, true or false.

The most obvious countermeasure to false beliefs and prejudices is a combination of rationality and education, possibly assisted by various other forms of cognitive enhancement, in addition to courses or sources of education and logic.^13^

Taken at face value, Harris' conclusion here is simply that the *most obvious* means to attenuating racial aversion are ones that operate by improving cognition. This is a very weak claim, and one that does not raise any serious worries about direct emotion modulation as a means to moral enhancement. Even if direct emotion modulation is not the most obvious means to this goal, its use could still be highly effective, morally permissible, and indeed morally desirable. However, Harris presents the passage as raising a ‘problem’ for noncognitive moral enhancement.^14^ Perhaps his thought is that the considerations that he appeals to here would also support a stronger conclusion: that *the only reasonably effective* means to attenuating racial aversion will operate improving cognition. Harris makes two different points that might be thought to support this claim. First, that racial aversion is likely to have ‘cognitive *content*’, for example because it is (partly) constituted by beliefs. And second, that racial aversion is likely to have cognitive causes, to be ‘*based on* false beliefs’.^15^ Harris' thought may be that the cognitive causes and content of racial aversion render it insusceptible to attenuation unless cognition-improving means are employed.

Harris may be right to point out that racial aversion is partly caused or constituted by cognitive states. If Ann is averse to Bob in virtue of Bob's race, Ann must, arguably, have some belief (if only a tacit one) about which racial group Bob belongs to. However, it is, I think, doubtful whether the cognitive causes or content of racial aversion must always be *false, stereotypic* racial beliefs of the sort that Harris seems to have in mind. Psychologists and neuroscientists study certain deeply ingrained fear- or disgust-based responses towards people of different race that seem aptly characterized as variants of racial aversion (they are often described as *implicit racial bias*) but which entail the presence of no false, stereotypic beliefs. Moreover, there seems little reason to suppose that such aversions must be *caused* by stereotypic beliefs. Indeed, recent psychological work suggests that the presence of implicit racial bias is uncorrelated with the possession of stereotypic racial beliefs, that the bias may be strong even in the relative absence of negative racial stereotypes, and that the bias and stereotypic beliefs motivate different behaviours.^16^ Meanwhile, results from neuroimaging studies suggest that implicit racial bias and stereotypic beliefs are mediated by distinct neural systems; the bias is associated with amygdala activity, which is also implicated in mediating fear and other basic emotions, while the stereotypic beliefs appear to be mediated by neural systems associated with other rational and cognitive processes.^17^ This suggests that there is at least one species of racial aversion – the kind studied by psychologists and neuroscientists in these studies – that is causally rather well-isolated from false, stereotypic beliefs.

More importantly, even where racial aversion does involve, or was caused by, false stereotypes, it might nevertheless be attenuated by means other than the correction of those faulty beliefs. There is no good reason to suppose that we can only effectively manipulate mental states by tackling their causes. Psychiatrists have many pharmaceutical means of treating depressed mood, but it is doubtful whether any of these tackle the causes of the depression. Similarly, painkillers can reduce pain without correcting its cause. Racial aversion, too, might be mitigated by means that leave its putative cognitive causes intact. Likewise, even if racial aversion is partly *constituted* by erroneous beliefs, it might be attenuated without correcting those beliefs. We might instead directly target the noncognitive elements of the aversion; for example, the physiological arousal that occurs when one is confronted with a person of different race.

That direct interventions might alter racial aversions, and other kinds of xenophobia, can be brought out by drawing a comparison with other sorts of phobia. Consider arachnophobia. Fearful responses to spiders may sometimes involve, or be caused by, certain false beliefs (for example, about the poisonousness of spiders). But even where this is so, arachnophobia can be treated via direct means. For example, fearful responses can be reduced by *systematic desensitisation*, in which the patient is repeatedly exposed to increasingly spider-like stimuli, though this need not correct any of the arachnophobic's false beliefs.^18^

If Harris is to accept that moral enhancement could consist in the attenuation of certain morally relevant emotions, then it is difficult to see how he could deny that it could be achieved through the direct modulation of those emotions. Even if the relevant emotions have cognitive content, and cognitive causes, we may still be able to attenuate them directly.

## THE SECOND CONCERN: RESTRICTION OF FREEDOM

In arguing against noncognitive moral enhancement, Harris appeals liberally to the famous passage from Milton's *Paradise Lost* in which God reports having ‘made [humans] just and right, sufficient to have stood, though free to fall’.^19^ However, it is not entirely clear that there is anything in this passage that should concern a proponent of noncognitive moral enhancement. If we read the claim that humans are ‘sufficient to have stood’ as implying that there is no *need* for moral enhancement – that humans already have sufficiently moral motives and behaviour – then it will look clearly false. It will also look inconsistent with Harris' own admission that we often succumb to temptation,^20^ and often have purposes other than, and in conflict with, moral goals.^21^ If, on the other hand, it is understood as holding merely that humans have the *capacity* for sufficiently moral motives and behaviour (henceforth, the capacity to be moral), then it seems quite consistent with the thought that noncognitive moral enhancement would be morally permissible and indeed desirable.

Why might noncognitive moral enhancement be desirable, even if we already possess the capacity to be moral? First, as Harris rightly notes, and I have previously emphasized, there are temptations and other factors that frequently prevent us from properly exercising this capacity. There is thus a clear scope for interventions that enhance our morality by directly mitigating those temptations and other counter-moral influences. There is a sense in which a recurrent sex offender with strong and deviant sexual desires has the capacity to behave morally. However, unless he is rightly confident that he will never again act on those desires, the offender should, plausibly, take steps to attenuate them.

Second, not everyone possesses the capacity for moral motivation and behaviour to the same degree. This suggests that there is scope for most people to strengthen or further develop this capacity, and it might often be a good thing if they did so. Emotions may be important here: they may influence the degree to which we possess the capacity for morality. This is particularly plausible if one thinks, as many have, that the capacity for moral motivation and behaviour requires certain conative or affective dispositions as well as cognitive capacities. Many have thought this. Aristotle can be understood as taking virtue to consist of certain emotional dispositions, acquired through habit earlier in life, and later fine tuned through the exercise of intellectual skills,^22^ and Hume and Mill took an ‘aversion to evil’ and ‘appetite to good’ to be central to moral motivation.^23^ However, those who take the moral capacity to be a purely rational one must also surely accept that emotional factors may still be relevant to its development. Even Immanuel Kant, who had a staunchly rational conception of human moral capacities, acknowledged a (limited) role for mechanical, noncognitive means of moral improvement, for example, via carrot and stick incentives. These could, he thought, be used to help instil discipline and other preconditions for the development of moral reasoning abilities.^24^ That Kant took this line is hardly surprising; emotions are relevant to the development of all sorts of rational capacities. Mathematical ability is a paradigmatically rational capacity, yet its acquisition depends on certain emotional influences, such as the extent to which one experiences pleasure when doing mathematics. Similarly, the development of moral reasoning skills presumably depends in part on the degree to which one enjoys engaging in such reasoning.

The view that humans typically already have a capacity to be moral is quite consistent with the view that it would be better if they faced fewer impediments to the exercise of this capacity (for example, temptations) and if they possessed the capacity to a higher degree. Direct emotion-modulating interventions might well help to achieve these aims.

Though Harris might deny, for reasons mentioned earlier, that direct emotion modulation would be an effective means to moral enhancement, he does acknowledge that there is scope, and perhaps a need, for such enhancement.^25^ Why, then, does he give such prominence to Milton's famous passage? The answer becomes evident towards the end of his article, when he writes that

part of Milton's insight is the crucial role of personal liberty and autonomy: that sufficiency to stand is worthless, literally morally bankrupt, without freedom to fall. … [M]y own view is that I, like so many others, would not wish to sacrifice freedom for survival. I might of course lack the courage to make that choice when and if the time comes. I hope however that I would, and I believe, on grounds that have more eloquently been so often stated by lovers of freedom throughout history, that freedom is certainly as precious, perhaps more precious than life.^26^

And then,

It is surely better to remain sufficient to stand and to hang on to our precious freedom to fall.^27^

The worry expressed here is not that our ‘sufficiency to stand’ renders noncognitive moral enhancement unnecessary or unimportant, but that our ‘freedom to fall’ renders it undesirable. Perhaps Harris believes that noncognitive moral enhancements would invariably restrict this freedom, depriving us of the option of having immoral motives, or behaving immorally. While immoral motives and conduct may themselves have little or no value, the *freedom* to hold such motives, or engage in such conduct, is, Harris suggests, highly valuable.

A similar thought underpins the well known free-will defence of theism, a staple response to the argument from evil. The argument from evil holds that there can be no omnipotent, omniscient and benevolent God, since that God would not allow evildoing to occur. The free will defence maintains, in reply, that evildoing is a consequence of our possessing the freedom to do evil, which is, all things considered, good. Though the freedom to do evil possesses the great instrumental disvalue of allowing evildoing, it also possesses some other, greater value.

If the freedom to do evil is all-things-considered valuable despite its very great instrumental disvalue, one might expect that the more general freedom to have immoral motives, or act immorally (henceforth, the freedom to be immoral), is also good despite *its* instrumental disvalue. Perhaps, then, noncognitive moral enhancement will be undesirable whenever it restricts that freedom.

However, it will not always do so. If I underwent some intervention that, say, gave me a powerful desire to give money to charity, I might have lost some of my freedom to be immoral. Perhaps this intervention can be construed as introducing a brute desire that restricts the freedom of my true self. However, now suppose that I undergo an intervention that mitigates some of the many emotional biases that afflict my prudential and moral reasoning, or that reduces my temptation to act against my sincere normative judgements. This intervention seems aptly characterized as *increasing* my freedom to be moral by removing a brute constraint on that freedom. At least, it is difficult to see how my freedom to be immoral has *decreased* by the change.

There is also the problem that, even where direct emotion modulation does decrease the freedom to be immoral, the moral cost of this may simply be outweighed by the moral benefit of improved motivation or behaviour. A standard response to the free will defence of theism holds that, in many cases, it seems preferable to sacrifice some freedom to do evil in order to prevent evil. If I witness one person about to murder another, it seems that I should intervene to prevent the murder even though this involves restricting the prospective murder's freedom to do evil. The obvious way of explaining this is by positing that, in this case at least, the freedom to do evil is less valuable than the evil is disvaluable. Similarly, there will presumably be cases of noncognitive moral enhancement where the disvalue of any loss in freedom to be immoral is outweighed by the value of the reduction in immoral behaviour or motivation.

## THE THIRD CONCERN: MORAL DECLINE

I now turn to Harris' third and perhaps most troubling concern. This is the concern that pursuing moral improvement through direct emotion modulation might in fact cause moral decline. My earlier suggestion was that, in some circumstances, a reduction in the extent to which a person experiences some emotion could qualify as a moral enhancement. But as Harris rightly notes, the emotion would have to be attenuated *to the right degree.* This is because, as Harris puts it, ‘the sorts of traits or dispositions that seem to lead to wickedness or immorality are also the very same ones required not only for virtue but for any sort of moral life at all’. I am not convinced that this applies to *all*‘dispositions to immorality’; it is not clear that xenophobia is, in normal circumstances, *at all* conducive to morality. However, it is undoubtedly true of some dispositions. Taken to excess, self-love, or love for one's immediate family and friends might typically impede moral motivation and behaviour, since they might lead one to be too self-interested, or too partial to one's immediate circle. But, some degree of self-love and love for one's kith and kin is surely conducive to morality. Similarly, a high degree of envy might often impede moral motivation and behaviour, but a lower level can be a spur to great (and morally good) achievements.

In a similar vein, Harris might have noted that attenuating some emotion might count as a moral enhancement in some *circumstances*, but not in others. Impulses to violent aggression, while often an impediment to morality, seem morally desirable in certain circumstances; for example, when one is fighting a just war, or perhaps when one is confronted with one person assaulting another on the street. Similarly, strong feelings of sympathy for victims of wrongdoing might impede morality in some circumstances – for example, when one is a judge charged with impartially hearing a criminal case – but will plausibly conduce to morality in others – for example, when one is in a good position to aid the victims of wrongdoing through charitable works.

Given these complications, it seems clear that what is necessary for moral enhancement is the *fine tuning* of certain emotions in a person-specific way that is sensitive to prevailing circumstances, not the wholesale elimination of emotions at a population level. Harris, for one, is doubtful that we could confidently achieve such fine tuning through direct emotion-modulating inventions; he doubts that we will ever have ‘precise and unequivocally good producing interventions’ of this sort. The concern here is not that moral enhancement through direct emotion modulation could never happen, but that, given the bluntness of the instruments (likely to be) available, it would rely on a good deal of luck. There will be a serious risk that any given attempt at moral enhancement will fail, perhaps resulting instead in moral decline.

This risk is certainly of concern. But does it constitute a decisive objection to attempting noncognitive moral enhancement? It is not clear that it does. Many medical interventions are risky in a similar way. Almost any medical intervention will, if pursued to too great a degree, have the opposite effect than that intended; it will *cause* health problems. Moreover, most medical interventions are rather blunt, and it is thus difficult to prevent them from having overall negative effects in some cases (this is perhaps particularly true of psychiatric interventions). However, we do not, and should not, regard this as providing us with decisive reasons to abstain from medical treatments. Rather, we take it as giving us reasons to exercise caution in using such treatments, and to try to reduce the risks posed by the treatments over time, for example, by making them more precise.

Perhaps, however, Harris' thought was not merely that attempts at noncognitive moral enhancement *could* result in moral decline, but that, as a matter of fact, they *typically will*. This would be an interesting result; it might in turn support the view that we should *typically* abstain from noncognitive moral enhancement. It might even justify a general discouragement or prohibition of noncognitive moral enhancement.

There are at least two factors that lend some support to the thought that attempts at noncognitive moral enhancement will typically lead to moral decline. First, it may be that techniques for the direct modulation of emotions will often be used recklessly or over-enthusiastically, without proper regard for the risk of moral decline. Second, they may frequently be used in the service of incorrect moral beliefs. Heinrich Himmler believed that morality required the extermination of the Jews, and thus the suppression of feelings of sympathy for them.^28^ In his eyes, the suppression of sympathy for Jews might have constituted a moral improvement, though it was in fact a moral deterioration. If seriously wrongheaded moral beliefs are the norm, then we might expect that most attempts at noncognitive moral enhancement will in fact lead in the wrong direction. (Here, I suppose that moral improvement would still be the goal, it would simply be frustrated by incorrect beliefs about what constitutes moral improvement. A more common problem, and one that I have previously noted, is likely to be that techniques for altering morally significant emotions will be used in the service of other goals. They might, for example, be used to intentionally induce immoral motives or behaviour in order, say, to achieve personal gain.^29^ However, in what follows I will, like Harris, focus on direct emotion modulation that at least *aims at* moral improvement.)

The concern that attempts at noncognitive moral enhancement would typically result in moral decline should, I think, be taken seriously. There is, after all, a worrying history of reckless or misguided attempts at noncognitive moral enhancement. Psychiatric or neuroscientific means of directly influencing emotions, without improving cognition have often been used without proper regard for the subtleties of brain function, and in the service of mistaken moral beliefs. The history of frontal lobotomy and other psychosurgery is informative.^30^ Another example comes from the use of brain implants capable of electrically stimulating the brain. In 1969, the Yale physiologist José Delgado published *Physical Control of the Mind: Toward a Psychocivilised Society* in which he described his work modifying behaviour in both humans and animals via electrical brain stimulation.^31^ In perhaps the most graphic demonstration of his techniques, he used brain stimulation to halt a charging bull just a few feet from impaling him. In another experiment, he induced sudden rage in a woman calmly playing the guitar. Delgado hoped that his brain stimulation techniques could be used to create a ‘less cruel, happier, and better man’,^32^ but within a matter of months, other scientists were defending the use of brain stimulation to quell civil rights riots.^33^ The techniques were also later used in attempts to ‘cure’ homosexuality.^34^

Not all past and present attempts at noncognitive moral enhancement have been so problematic, however. Arguably, early childhood discipline that punishes harmdoing and rewards sharing and co-operation often constitutes an attempt at noncognitive moral enhancement. Yet it is debatable whether it typically induces moral decline. Similarly, stimulus avoidance has been used as a form of noncognitive moral enhancement by some people with aggressive or other anti-social tendencies, and again, it is unclear that it has primarily been a force for moral decline.

Overall, then, it seems unclear whether experience to date should lead us to reject noncognitive moral enhancement. Perhaps it can be shown that attempts at noncognitive moral enhancement have typically had, or typically do have, negative moral effects. But this is not already obvious. Demonstrating it would seem to require an in-depth historical study that has not yet been undertaken. Alternatively, perhaps it can be argued that history is a misleading guide – that even if attempts at noncognitive moral enhancement have generally turned out well to date, future attempts will typically have negative moral effects. Perhaps future techniques for noncognitive moral enhancement will be more liable to reckless or over-enthusiastic use than existing ones. Or perhaps future people will be more disposed to recklessness and over-enthusiasm than we are. However, here too, further argument seems called for. There is surely at least a *prima facie* reason to suppose that the future will mirror the past and present.

The position I have reached, then, is as follows. Harris is right to point out that if noncognitive moral enhancement is to be achieved through the direct modulation of emotions, then what is called for is the *fine-tuning* of those emotions. He is also right to note that existing and likely near-term future means to noncognitive moral enhancement are rather blunt. There is therefore a risk that attempts at such enhancement will in fact lead to moral decline. However, the mere presence of a risk of moral decline does not show that we have decisive reasons against attempting noncognitive moral enhancement; reasons to avoid that risk might well be matched or outweighed by other, countervailing reasons. Harris could, of course, argue that attempts at moral enhancement will *typically* lead to moral decline. But he does not himself demonstrate this. Further argument, and perhaps a detailed historical study of noncognitive moral enhancement, would be required to establish this point.

## CONCLUSIONS

As I have interpreted him, Harris raises the following three concerns about noncognitive moral enhancement: that direct intervention in emotional states will be ineffective as a means to moral enhancement, that noncognitive moral enhancement involves problematic restrictions on freedom, and that attempts at noncognitive moral enhancement may end up causing moral decline. The first concern is, I have argued, misplaced. The claims Harris uses to support it – that morally relevant emotions have cognitive content, and that they have cognitive causes – do nothing to show that these emotions could not be modulated directly. On the other hand, there is, I think, something to each of the other concerns. Perhaps emotional enhancements could, in some cases, restrict a valuable freedom – the freedom to hold moral motives, or to behave immorally. And they could, particularly if used recklessly or in the service of incorrect moral beliefs, cause moral decline. But I do not think these concerns support any more than the rather weak conclusion that, *sometimes*, attempting or engaging in noncognitive moral enhancement will be impermissible. It might be possible to further develop the worry about moral decline in order to defend a more troubling conclusion; perhaps it could be argued that attempts at noncognitive moral enhancement will *typically* result in moral decline. But this is an argument that Harris does not provide.

## Footnotes

1 See, for example, H. Arendt. 1963. *Eichmann in Jerusalem: A Report on the Banality of Evil*. New York: Viking; S. Milgram. 1974. *Obedience to Authority: An Experimental View*. London: Tavistock; R.J. Lifton. 1986. *The Nazi Doctors: Medical Killing and the Psychology of Genocide*. New York: Basic Books; E. Staub. 1989. *The Roots of Evil: The Origins of Genocide and Other Group Violence*. New York: Cambridge University Press; J.M. Darley. Social Organization for the Production of Evil. *Psychol Inq* 1992; 3(2): 199–218; J. Glover. 1999. *Humanity: A Moral History of the Twentieth Century*. London: Jonathan Cape.

2 Glover, *op. cit*. note 1.

3 See, for example, A. Buchanan. Institutions, Beliefs and Ethics: Eugenics as a Case Study. J Polit Philos 2007; 15(1): 22–45; A. Buchanan. Philosophy and Public Policy: A Role for Social Moral Epistemology. J Appl Philos 2009; 26(3): 276–290; J. Bakan. 2004. The Corporation: The Pathological Pursuit of Profit and Power. New York: Free Press.

4 I. Persson & J. Savulescu. The Perils of Cognitive Enhancement and the Urgent Imperative of Enhance the Moral Character of Humanity. *J Appl Philos* 2008; 25(3): 162–177; I. Persson & J. Savulescu. 2011. Unfit for the Future? Human Nature, Scientific Progress, and the Need for Moral Enhancement. In: *Enhancing Human Capabilities*. J. Savulescu, R. ter Meulen, G. Kahane, eds. Oxford: Wiley-Blackwell.

5 T. Douglas. Moral Enhancement. *J Appl Philos* 2008; 25(3): 228–245. See also H.S. Faust. Should We Select for Genetic Moral Enhancement? A Thought Experiment Using the MoralKinder (MK+) Haplotype. *Theor Med Bioeth* 2008; 29(6): 397–416.

6 Glover, *op. cit.* note 1, pp. 41–42; Persson & Savulescu, *op. cit*. note 4.

7 J. Riis,J.P. Simmons & G.P. Goodwin. Preferences for Enhancement Pharmaceuticals: The Reluctance to Enhance Fundamental Traits. *J Consum Res* 2008; 35(3): 495–508.

8 F. Fukuyama. 2002. *Our Posthuman Future: Consequences of the Biotechnology Revolution*. New York: Farrar, Straus and Giroux: 47–56; President's Council on Bioethics. 2003. *Beyond Therapy*: *Biotechnology and the Pursuit of Happiness*. Washington, DC: President's Council on Bioethics: 71–94.

9 Riis (*op. cit*. note 7) found that participants in their study were in general more reluctant to enhance traits that were also judged central to the self-identity.

10 J. Harris. Moral Enhancement and Freedom. *Bioethics* 2011: 25(2): 102–111, 104.

11 Harris, *op. cit*. note 10, pp. 103–105.

12 This deviates from my earlier formulation only in allowing that moral enhancement could consist in the moral improvement of behaviour even where there is no moral improvement in motives.

13 Harris, *op. cit*. note 10, pp. 104–105.

14 Ibid: 104.

15 Ibid: 105. My italics.

16 D.M. Amodio & P.G. Devine. Stereotyping and Evaluation in Implicit Race Bias: Evidence for Independent Constructs and Unique Effects on Behaviour. *J Pers Soc Psychol* 2006; 91: 652–661; D.M. Amodio. The Social Neuroscience of Intergroup Relations. *Eur Rev Soc Psychol* 2008; 19(1): 1–54: 7–18.

17 Amodio, *op. cit*. note 16, pp. 16–18.

18 Ingmar Persson suggested this example to me.

19 J. Milton. 2000. *Paradise Lost*. J. Leonard, ed. London: Penguin Books.

20 Harris, *op. cit*. note 10, pp. 103–104.

21 Ibid: 104.

22 See Aristotle. 1984. Nicomachean Ethics. In *The Complete Works of Aristotle* Vol II. Revised edn. J. Barnes, ed. Princeton, NJ: Princeton University Press: 1729–1867, Book II 1742–1743 [1103], Book III 1759, 1767 [1114, 1119]. Page numbers in square brackets refer to I. Bekker's 1831 Greek edition of Aristotle's collected works.

23 The quotes are from D. Hume. 1978. *A Treatise of Human Nature*. Second edn. L.A. Selby-Bigge, ed. Oxford: Clarendon Press: Book II 417. John Stuart Mill refers to ‘a feeling in our own mind; a pain, more or less intense, attendant on violation of duty, which in properly cultivated moral natures rises, in the more serious cases into shrinking from it as an impossibility’. See J.S. Mill. 1979. *Utilitarianism*. Indianapolis: Hackett: 27.

24 I. Kant. 2008. *The Critique of Practical Reason*. T.K. Abbott, trans. Charleston, SC: Forgotten Books: 130, 137 [152, 160]; G.F. Munzel. Kant on Moral Education, or ‘Enlightenment’ and the Liberal Arts. *Rev Metaphys* 2003; 57(1): 43–73: 65–66. Page numbers in square brackets are for the Prussian Academy edition of Kant's collected works.

25 Harris, *op. cit*. note 10, pp. 103–104, 110.

26 Ibid: 110–111.

27 Ibid: 111.

28 J. Bennett. The Conscience of Huckleberry Finn. *Philosophy* 1974; 49(188): 123–134.

29 Douglas, *op. cit*. note 5.

30 H.T. Greely. Neuroscience and Criminal Justice: Not Responsibility but Treatment. *Kansas Law Review* 2008; 56(5): 1103–1138.

31 J.M.R. Delgado. 1969. *Physical Control of the Mind: Toward a Psychocivilized Society*. New York: Harper & Row. See also J.A. Osmundsen. 1965. ‘Matador’ with a Radio Stops Wired Bull: Modified Behaviour in Animals the Subject of Brain Study. *New York Times* 17 May. Available at http://www.wireheading.com/matador.html[accessed 23 Nov 2010]; J. Horgan. The Forgotten Era of Brain Chips. *Sci Am* 2005; 293(4): 66–73. I thank Andrew Jackson for drawing my attention to Delgado's work.

32 Delgado, *op. cit*. note 30, cited in Horgan, *op. cit*. note 30, p. 71.

33 V.H. Mark & F.R. Ervin. 1970. *Violence and the Brain*. New York: Harper & Row; Horgan, *op. cit*. note 30, p. 71.

34 Horgan, *op. cit*. note 30, p. 71.

## Notes

**Thomas Douglas** is a Wellcome Trust Research Fellow at the Oxford Uehiro Centre for Practical Ethics and a Junior Research Fellow at Balliol College, Oxford. He trained in both medicine and philosophy and has written on the ethics of biomedical enhancement, slippery slope arguments, compensatory justice, organ donation and reproductive ethics.

